# Prevalence of Multiple Chronic Conditions Among Medicare Beneficiaries, United States, 2010

**DOI:** 10.5888/pcd10.120137

**Published:** 2013-04-25

**Authors:** Kimberly A. Lochner, Christine S. Cox

**Affiliations:** Author Affiliation: Christine S. Cox, Centers for Medicare and Medicaid Services, Baltimore, Maryland.

## Abstract

**Introduction:**

The increase in chronic health conditions among Medicare beneficiaries has implications for the Medicare system. The objective of this study was to use the US Department of Health and Human Services Strategic Framework on multiple chronic conditions as a basis to examine the prevalence of multiple chronic conditions among Medicare beneficiaries.

**Methods:**

We analyzed Centers for Medicare and Medicaid Services administrative claims data for Medicare beneficiaries enrolled in the fee-for-service program in 2010. We included approximately 31 million Medicare beneficiaries and examined 15 chronic conditions. A beneficiary was considered to have a chronic condition if a Medicare claim indicated that the beneficiary received a service or treatment for the condition. We defined the prevalence of multiple chronic conditions as having 2 or more chronic conditions.

**Results:**

Overall, 68.4% of Medicare beneficiaries had 2 or more chronic conditions and 36.4% had 4 or more chronic conditions. The prevalence of multiple chronic conditions increased with age and was more prevalent among women than men across all age groups. Non-Hispanic black and Hispanic women had the highest prevalence of 4 or more chronic conditions, whereas Asian or Pacific Islander men and women, in general, had the lowest.

**Conclusion:**

The prevalence of multiple chronic conditions among the Medicare fee-for-service population varies across demographic groups. Multiple chronic conditions appear to be more prevalent among women, particularly non-Hispanic black and Hispanic women, and among beneficiaries eligible for both Medicare and Medicaid benefits. Our findings can help public health researchers target prevention and management strategies to improve care and reduce costs for people with multiple chronic conditions.

## Introduction

The increase in chronic health conditions among Medicare beneficiaries has far-reaching implications for the Medicare system (1,2). Among Medicare beneficiaries, not only are hypertension, high cholesterol, heart disease, and diabetes highly prevalent, but most beneficiaries have multiple chronic conditions. Medicare beneficiaries with multiple chronic conditions are the heaviest users of health care services, including such high-cost services as hospitalizations and emergency department visits, which translates into increased Medicare spending. For example, the two-thirds of beneficiaries with 2 more chronic conditions accounted for 93% of Medicare spending, and the one-third with 4 or more chronic conditions accounted for almost three-fourths of Medicare spending (3).

Although research has focused on chronic conditions such as hypertension, diabetes, and heart conditions (1), the US Department of Health and Human Services (HHS) Initiative on multiple chronic conditions calls for the need to enhance the understanding of chronic condition comorbidities. The HHS Initiative’s strategic framework on multiple chronic conditions offers an organizing structure to address multiple chronic conditions through research, interventions, and health care management and indicates the need to increase the evidence base on the epidemiology of multiple chronic conditions (4,5).

The objective of this study was to use the HHS Strategic Framework on multiple chronic conditions as a basis to examine the prevalence of multiple chronic conditions among Medicare beneficiaries. In combination with the other articles in this issue of *Preventing Chronic Disease* that address multiple chronic conditions, this study begins to fill the gaps and improve our understanding of the prevalence of multiple chronic conditions across different populations using different data sources. 

## Methods

Medicare is the US federal health insurance program for people aged 65 or older, people younger than 65 with certain disabilities, and people of any age with end-stage renal disease. We examined Centers for Medicare and Medicaid Services (CMS) administrative enrollment and claims data for Medicare beneficiaries enrolled in the fee-for-service program for 2010. These CMS data are available from the CMS Chronic Condition Data Warehouse (CCW), a research database with 100% of Medicare enrollment and fee-for-service claims data that is designed to make CMS data more readily available to support research. (6). 

In 2010, there were more than 50 million Medicare beneficiaries. The study population included only those Medicare beneficiaries continuously enrolled in Medicare fee-for-service parts A and B, also known as original or traditional Medicare, for 2010. To obtain a study population of beneficiaries continuously enrolled in fee-for-service Medicare for the entire year, we excluded beneficiaries with any Medicare Advantage enrollment during the year, approximately 17 million beneficiaries, and beneficiaries who first became eligible for Medicare after January of the calendar year. We included beneficiaries who died during the year up to their date of death if they met other inclusion criteria. Our 2010 Medicare fee-for-service study population comprised approximately 31 million beneficiaries and represented approximately 66% of the total Medicare population in 2010.

We included the following 15 chronic conditions in our examination of multiple chronic conditions: Alzheimer’s disease and related dementia, arthritis, asthma, atrial fibrillation, cancer (breast, colorectal, lung, prostate), chronic kidney disease, chronic obstructive pulmonary disease, depression, diabetes, heart failure, hyperlipidemia, hypertension, ischemic heart disease, osteoporosis, and stroke. We considered Medicare beneficiaries to have a chronic condition if the CMS administrative data had a claim indicating that beneficiaries received a service or treatment for the condition. For example, to identify a beneficiary with hyperlipidemia during 2010, at least 1 inpatient, skilled nursing facility, or home health claim or 2 outpatient or carrier claims had to include any of the following diagnosis codes from the *International Classification of Diseases, 9th Revision, Clinical Modification*: 272.0, 272.1, 272.2, 272.3, 272.4. Detailed information on the identification of chronic conditions in the CCW is available elsewhere (6). Chronic conditions were counted and grouped into 3 categories (0 or 1, 2 or 3, and 4 or more); multiple chronic conditions were defined as having 2 or more chronic conditions. We also identified the most common dyads of chronic conditions among beneficiaries with at least 2 chronic conditions, as well as the most common triads among beneficiaries with at least 3 chronic conditions.

We examined the treated prevalence of multiple chronic conditions by select Medicare beneficiary characteristics: sex, age in years (<65, 65–74, 75–84, and ≥85), dual Medicaid enrollment, also known as “dual eligible,” and race/ethnicity (non-Hispanic white, non-Hispanic black, Hispanic, Asian or Pacific Islander, American Indian or Alaska Native, and non-Hispanic other race.). Because of known limitations in Medicare enrollment data for the classification of a beneficiary’s race/ethnicity, we used the race/ethnicity variable available in the CCW that is based on a validated algorithm that improves the accuracy of race/ethnicity classification (7).

## Results

Among our population of fee-for-service Medicare beneficiaries, 17.1% percent were younger than 65, who receive Medicare primarily as a result of having a disability, and 82.9% were aged 65 or older (Table 1). Female beneficiaries were older than male beneficiaries, particularly in the group aged 85 or older (17.8% of women compared with 10.7% of men). Our study population was 81.2% non-Hispanic white, 9.6% non-Hispanic black, 5.7% Hispanic, and 2.2% Asian or Pacific Islander. Race/ethnicity did not vary greatly between men and women. Approximately one-fifth (21.6%) of Medicare beneficiaries were also eligible for Medicaid; more female beneficiaries were dual-eligible than men.

**Table 1:** Study Population Characteristics for Medicare Fee-for-Service Beneficiaries, 2010

Characteristic	Total, % (N = 31,313,331)	Men, % (n = 13,845,487)	Women, % (n = 17,467,844)
**Age, y**			
<65	17.1	20.4	14.6
≥65	82.9	79.7	85.4
65–74	39.3	41.3	37.6
75–84	29.0	27.7	30.0
≥85	14.6	10.7	17.8
**Sex**			
Male	44.2	NA	NA
Female	55.8	NA	NA
**Race/ethnicity**			
Non-Hispanic white	81.2	81.0	81.3
Non-Hispanic black	9.6	9.4	9.8
Hispanic	5.7	6.0	5.5
Non-Hispanic Asian/Pacific Islander	2.2	2.1	2.2
Non-Hispanic American Indian/Alaska Native	0.5	0.5	0.5
Non-Hispanic other race	0.9	1.0	0.8
**Dual-eligibility** a			
Yes	21.6	19.1	23.7
No	78.4	80.9	76.3

Abbreviation: NA, not applicable.

a Refers to Medicare beneficiaries who also were eligible for Medicaid.

Overall, 68.4% of Medicare beneficiaries had 2 or more chronic conditions and 36.4% had 4 or more chronic conditions (Table 2). The prevalence of multiple chronic conditions (≥2) increased with age and was higher for women than men across all age groups. Among beneficiaries younger than 65, 48.7% of men had multiple chronic conditions compared with 58.8% of women. In this age group for both men and women, the prevalence of multiple chronic conditions was highest for non-Hispanic blacks and Hispanics (approximately 50% for men and 60%–64% for women) and lowest for Asian/Pacific Islanders (46.8% for men and 50.3% for women). Among beneficiaries 65 and older, 69.1% of men had multiple chronic conditions compared with 73.4% of women. Among men 65 or older, the highest prevalence of multiple chronic conditions was among non-Hispanic whites (69.6%) and the lowest among Hispanics (63.1%), but among women in this age group, the highest prevalence was among non-Hispanic blacks (79.6%) and the lowest among Asian/Pacific Islanders and non-Hispanic whites. When we compared the prevalence of 4 or more chronic conditions with the prevalence of 2 or more, the patterns were similar for beneficiaries younger than 65 but varied for beneficiaries 65 or older (Table 2).

**Table 2:** Percentage of Medicare Fee-for-Service Beneficiaries With Chronic Conditions, by Sex, Age, and Race/Ethnicity, 2010

Characteristic	No. of Chronic Conditions		

	0 or 1	2 or 3	≥4
**Total (N = 31,313,331)**	31.5	32.0	36.4
**Men**			
**Aged <65 (n = 2,817,692)**	51.3	26.0	22.7
Non-Hispanic white	51.9	26.3	21.9
Non-Hispanic black	50.1	25.2	24.8
Hispanic	49.4	25.6	25.0
Non-Hispanic Asian/Pacific Islander	53.2	25.4	21.4
Non-Hispanic American Indian/Alaska Native	52.2	25.7	22.1
Non-Hispanic other race	51.0	26.3	22.7
**Aged ≥65 (n = 11,027,795)**	30.9	31.2	37.9
Non-Hispanic white	30.4	31.7	37.9
Non-Hispanic black	31.5	28.3	40.2
Hispanic	36.9	26.0	37.1
Non-Hispanic Asian/Pacific Islander	32.1	33.0	34.9
Non-Hispanic American Indian/Alaska Native	33.1	29.8	37.1
Non-Hispanic other race	37.8	30.9	31.3
**Women**			
**Aged <65 (n = 2,548,598)**	41.2	30.3	28.5
Non-Hispanic white	42.7	30.5	26.9
Non-Hispanic black	36.0	30.2	33.8
Hispanic	39.8	29.1	31.0
Non-Hispanic Asian/Pacific Islander	49.6	28.7	21.6
Non-Hispanic American Indian/Alaska Native	41.5	30.2	28.3
Non-Hispanic other race	46.8	29.4	23.8
**Aged ≥65 (n = 14,919,246)**	26.6	34.1	39.3
Non-Hispanic white	27.2	34.5	38.3
Non-Hispanic black	20.5	32.9	46.7
Hispanic	24.8	28.9	46.4
Non-Hispanic Asian/Pacific Islander	27.8	35.3	36.9
Non-Hispanic American Indian/Alaska Native	27.6	33.0	39.4
Non-Hispanic other race	34.3	33.1	32.6

The prevalence of multiple chronic conditions, in particular 4 or more chronic conditions, increased with age ( Table 3). Non-Hispanic black men had the highest prevalence of 4 or more chronic conditions among men aged 65 to 74 and 75 to 84 (34.2% and 46.9%, respectively). For men 85 or older, non-Hispanic whites, non-Hispanic blacks and Hispanics had similar rates of 4 or more chronic conditions (54.6%). For women across all older age groups, non-Hispanic black and Hispanic women had the highest prevalence of 4 or more chronic conditions.

**Table 3:** Percentage of Medicare Fee-for-Service Beneficiaries Aged ≥65 With Chronic Conditions, by Sex, Expanded Age, and Race/Ethnicity, 2010

Characteristic	No. of Chronic Conditions		

	0 or 1	2 or 3	≥4
**Total (n = 25,947,041)**	28.4	32.9	38.7
**Men**			
**Aged 65–74 (n = 5,719,126)**	38.4	32.5	29.0
Non-Hispanic white	38.2	33.2	28.6
Non-Hispanic black	36.3	29.5	34.2
Hispanic	43.4	27.0	29.6
Non-Hispanic Asian/Pacific Islander	40.2	34.0	25.8
Non-Hispanic American Indian/Alaska Native	37.7	30.3	32.0
Non-Hispanic other race	42.0	31.7	26.3
**Aged 75–84 (n = 3,830,486)**	24.4	30.8	44.8
Non-Hispanic white	24.0	31.3	44.7
Non-Hispanic black	25.6	27.4	46.9
Hispanic	29.9	25.4	44.7
Non-Hispanic Asian/Pacific Islander	24.7	33.4	42.0
Non-Hispanic American Indian/Alaska Native	27.3	29.5	43.2
Non-Hispanic other race	28.0	30.2	41.8
**Aged ≥85 (n = 1,478,183)**	18.6	27.0	54.5
Non-Hispanic white	18.1	27.4	54.6
Non-Hispanic black	21.4	24.0	54.6
Hispanic	23.5	21.9	54.6
Non-Hispanic Asian/Pacific Islander	20.4	28.3	51.3
Non-Hispanic American Indian/Alaska Native	23.8	27.5	48.7
Non-Hispanic other race	27.1	23.5	49.4
**Women**			
**Aged 65–74 (n = 6,571,342)**	35.5	35.8	28.7
Non-Hispanic white	36.8	36.1	27.1
Non-Hispanic black	25.8	35.4	38.8
Hispanic	31.4	31.4	37.3
Non-Hispanic Asian/Pacific Islander	35.7	37.2	27.1
Non-Hispanic American Indian/Alaska Native	33.0	33.8	33.2
Non-Hispanic other race	40.5	34.7	24.8
**Aged 75–84 (n = 5,247,284)**	21.5	34.5	44.0
Non-Hispanic white	22.1	35.1	42.8
Non-Hispanic black	16.2	32.0	51.7
Hispanic	19.0	27.5	53.5
Non-Hispanic Asian/Pacific Islander	21.5	34.8	43.7
Non-Hispanic American Indian/Alaska Native	22.9	32.7	44.4
Non-Hispanic other race	26.5	33.4	40.2
**Aged ≥85 (n = 3,100,620)**	16.3	30.0	53.7
Non-Hispanic white	16.4	30.5	53.1
Non-Hispanic black	13.7	27.6	58.7
Hispanic	15.7	23.5	60.8
Non-Hispanic Asian/Pacific Islander	18.7	30.7	50.6
Non-Hispanic American Indian/Alaska Native	18.9	30.5	50.7
Non-Hispanic other race	26.5	25.9	47.6

In general, dual-eligible beneficiaries had a higher prevalence of multiple chronic conditions than nondual beneficiaries, and female dual-eligible beneficiaries had a higher prevalence than male dual-eligible beneficiaries at every age (Table 4). Among female dual-eligible beneficiaries, the prevalence of multiple chronic conditions was 81.2% for those aged 65 to 74, 88.0% for those aged 75 to 84, and 91.7% for those aged 85 or older. The prevalence of 4 or more chronic conditions was high for this vulnerable population; approximately two-thirds of beneficiaries that were dual-eligible and aged 85 or older (both men and women) had 4 or more chronic conditions.

**Table 4:** Percentage of Medicare Fee-for-Service Beneficiaries With Chronic Conditions by Sex, Age, and Dual-Eligibility Status^a^ (N = 31,313,331), 2010

Characteristic	No. of Chronic Conditions		

	0 or 1	2 or 3	≥ 4
Men			
**Aged <65**	51.3	26.0	22.7
Dual	49.2	27.1	23.7
Nondual	53.6	24.8	21.6
**Aged 65–74**	38.4	32.5	29.0
Dual	26.9	27.3	45.8
Nondual	39.8	33.1	27.1
**Aged 75–84**	24.4	30.8	44.8
Dual	18.2	24.0	57.8
Nondual	25.2	31.6	43.2
**Aged ≥85**	18.6	27.0	54.5
Dual	11.5	21.1	67.4
Nondual	19.6	27.8	52.6
**Women**			
**Aged <65**	41.2	30.3	28.5
Dual	38.3	30.4	31.3
Nondual	45.4	30.0	24.5
**Aged 65–74**	35.5	35.8	28.7
Dual	18.7	30.0	51.2
Nondual	38.4	36.8	24.8
**Aged 75–84**	21.5	34.5	44.0
Dual	12.0	25.9	62.1
Nondual	23.6	36.3	40.2
**Aged ≥85**	16.3	30.0	53.7
Dual	8.3	24.1	67.6
Nondual	18.9	32.0	49.2

a Dual eligibility refers to beneficiaries that are eligible to receive benefits under both the Medicare and Medicaid programs.

Among beneficiaries with 2 or more chronic conditions, the most prevalent dyads included hypertension, hyperlipidemia, diabetes, and ischemic heart disease (Table 5). The most common dyad across all sex and age groups was hypertension and hyperlipidemia; however, among beneficiaries younger than 65, the diabetes and hyperlipidemia was also highly prevalent. Women differed from men in that depression and arthritis were present in the most common dyads. We examined triads of chronic conditions only for beneficiaries who had at least 3 chronic conditions. For beneficiaries younger than 65, the most common triad of chronic conditions was diabetes, hypertension, and hyperlipidemia; 35.4% of men and 32.0% of women had these 3 conditions (Table 6). Among beneficiaries 65 years or older, ischemic heart disease, hypertension, and hyperlipidemia was a common triad; 42.6% of men and 29.4% of women had these 3 conditions.

**Table 5:** Five Most Prevalent Chronic Condition Dyads Among Medicare Fee-for-Service Beneficiaries, by Sex and Age Group (N = 21,437,857), 2010^a^

Dyad by Sex and Age Group	Prevalence, %
**Men**	
**<65**	
Hypertension and hyperlipidemia	45.2
Diabetes and hyperlipidemia	37.9
Ischemic heart disease and hyperlipidemia	31.0
Diabetes and hypertension	29.6
Ischemic heart disease and hypertension	24.6
**≥65**	
Hypertension and hyperlipidemia	56.0
Ischemic heart disease and Hyperlipidemia	44.6
Ischemic heart disease and hypertension	39.0
Diabetes and hyperlipidemia	33.9
Diabetes and hypertension	28.6
**Women**	
**<65**	
Hypertension and hyperlipidemia	39.9
Diabetes and hyperlipidemia	35.0
Depression and hyperlipidemia	29.7
Arthritis and hyperlipidemia	29.4
Diabetes and hypertension	27.5
**≥65**	
Hypertension and hyperlipidemia	53.5
Arthritis and hyperlipidemia	36.8
Ischemic heart disease and hyperlipidemia	32.9
Diabetes and hyperlipidemia	30.0
Arthritis and hypertension	26.7

a Medicare beneficiaries had to have at least 2 of the chronic conditions listed.

**Table 6:** Five Most Prevalent Chronic Condition Triads Among Medicare Fee-for-Service Beneficiaries, by Sex and Age Group (N = 16,481,558), 2010^a^

Triad by Sex and Age Group	Prevalence, %
**Men**	
**<65**	
Diabetes, hypertension, and hyperlipidemia	35.4
Ischemic heart disease, hypertension, and hyperlipidemia	30.1
Diabetes, ischemic heart disease, and hyperlipidemia	25.3
Diabetes, ischemic heart disease, and hypertension	20.1
Diabetes, chronic kidney disease, and hyperlipidemia	18.8
**≥65**	
Ischemic heart disease, hypertension, and hyperlipidemia	42.6
Diabetes, hypertension, and hyperlipidemia	32.1
Diabetes, ischemic heart disease, and hyperlipidemia	25.9
Diabetes, ischemic heart disease, and hypertension	22.3
Arthritis, hypertension, and hyperlipidemia	21.8
**Women**	
**<65**	
Diabetes, hypertension, and hyperlipidemia	32.0
Arthritis, hypertension, and hyperlipidemia	23.5
Depression, hypertension, and hyperlipidemia	23.1
Ischemic heart disease, hypertension, and hyperlipidemia	21.9
Diabetes, arthritis, and hyperlipidemia	19.9
**≥65**	
Arthritis, hypertension, and hyperlipidemia	29.6
Ischemic heart disease, hypertension, and hyperlipidemia	29.4
Diabetes, hypertension, and hyperlipidemia	27.6
Ischemic heart disease, arthritis, and hyperlipidemia	20.8
Diabetes, ischemic heart disease, and hyperlipidemia	18.3

a Medicare beneficiaries had to have at least 3 of the chronic conditions listed.

## Discussion

The magnitude of CMS data allowed us to examine multiple chronic conditions by relevant sociodemographic characteristics to identify the Medicare beneficiaries who may benefit most from targeted interventions and health care management strategies aimed at delivering health care in a more effective and efficient manner. More than 21 million Medicare fee-for-service beneficiaries had 2 or more chronic conditions in 2010, and more than 11 million had 4 or more. Our findings support the goals and objectives of the HHS Strategic Framework on multiple chronic conditions, specifically the fourth goal that addresses the need for research to fill knowledge gaps about the epidemiology of multiple chronic conditions (4). Our findings also demonstrate the variation in multiple chronic conditions across demographic groups. Because the number of multiple chronic conditions increases with age and because women generally live longer than men do, the prevalence of multiple chronic conditions is expected to be higher for women. We found that female beneficiaries had a higher prevalence of multiple chronic conditions than male beneficiaries across all age groups. We also found that the prevalence of multiple chronic conditions varied by race/ethnicity. Non-Hispanic black and Hispanic women often had the highest prevalence of multiple chronic conditions. Multiple chronic conditions were more prevalent among the vulnerable population of dual-eligible beneficiaries, who tend to have low incomes or disabilities and be aged 85 or older.

Other studies may have included a larger number and broader set of chronic conditions to estimate the prevalence of multiple chronic conditions (1,2,8). Our estimates were determined on the basis of guidelines established by the HHS Strategic Framework, which defines multiple chronic conditions as having 2 or more chronic conditions and identifies a proposed set of 20 common chronic conditions (9). Our study included 15 of the 20 proposed HHS conditions but excluded several behavioral and other illnesses that are included in the HHS Framework (ie, autism spectrum disorder, schizophrenia, substance abuse [drug and alcohol disorders], HIV, and hepatitis, as well as conditions considered chronic by other sources) (10).

Our study has limitations. We identified chronic conditions through administrative claims in which discrepancies in physician coding could have introduced errors or lack of treatment for a condition could have resulted in misclassification., Also, our findings are applicable to the Medicare fee-for-service population. The CCW does not contain managed care claims or encounter data; therefore, examining multiple chronic conditions among Medicare beneficiaries enrolled in Medicare Advantage plans was not possible. However, our study did include more than 31 million Medicare beneficiaries, including disabled beneficiaries and those dually eligible for Medicaid.

Multiple chronic conditions lead to poor health outcomes, the use of high-cost health services, and increased Medicare spending. Our findings should provide health policy makers and the public health research community a better understanding of the burden of chronic conditions among the Medicare fee-for-service population and provide preliminary insights into developing prevention and management strategies that will improve care and reduce costs for people with chronic conditions.
